# Cannabinoid Receptors CB1 and CB2 Modulate the Electroretinographic Waves in Vervet Monkeys

**DOI:** 10.1155/2016/1253245

**Published:** 2016-03-16

**Authors:** Joseph Bouskila, Vanessa Harrar, Pasha Javadi, Amy Beierschmitt, Roberta Palmour, Christian Casanova, Jean-François Bouchard, Maurice Ptito

**Affiliations:** ^1^School of Optometry, University of Montreal, Montreal, QC, Canada H3T 1P1; ^2^Biomedical Sciences, Faculty of Medicine, University of Montreal, Montreal, QC, Canada H3T 1J4; ^3^St. Kitts Behavioral Science Foundation, Basseterre, Saint Kitts and Nevis; ^4^Department of Human Genetics, McGill University, Montreal, QC, Canada H3A 1B1; ^5^BrainLab and Neuropsychiatry Laboratory, Department of Neuroscience and Pharmacology, University of Copenhagen, Copenhagen, Denmark

## Abstract

The expression patterns of the cannabinoid receptor type 1 (CB1R) and the cannabinoid receptor type 2 (CB2R) are well documented in rodents and primates. In vervet monkeys, CB1R is present in the retinal neurons (photoreceptors, horizontal cells, bipolar cells, amacrine cells, and ganglion cells) and CB2R is exclusively found in the retinal glia (Müller cells). However, the role of these cannabinoid receptors in normal primate retinal function remains elusive. Using full-field electroretinography in adult vervet monkeys, we recorded changes in neural activity following the blockade of CB1R and CB2R by the intravitreal administration of their antagonists (AM251 and AM630, resp.) in photopic and scotopic conditions. Our results show that AM251 increases the photopic a-wave amplitude at high flash intensities, whereas AM630 increases the amplitude of both the photopic a- and b-waves. In scotopic conditions, both blockers increased the b-wave amplitude but did not change the a-wave amplitude. These findings suggest an important role of CB1R and CB2R in primate retinal function.

## 1. Introduction

The endocannabinoid system is composed of cannabinoid receptor type 1 (CB1R), cannabinoid receptor type 2 (CB2R), their endogenous ligands (endocannabinoids), and their synthetizing and metabolizing enzymes. The physiological and psychological effects of cannabinoids can be detected almost everywhere in the body due to the abundance of cannabinoid receptors. Expression patterns of CB1R and CB2R are well documented in the retina of numerous species, including rodents and primates [1–6]. In rodents, CB1R and CB2R are expressed in many retinal cell types, particularly cone and rod photoreceptors, horizontal cells, amacrine cells, bipolar cells, and ganglion cells [1, 7]. In vervet monkeys, CB1R is mainly found in cones of the central retina, rod spherules with very low expression, horizontal cells, bipolar cells, and amacrine and ganglion cells [5]. CB2R, on the other hand, is strictly expressed in primate glial Müller cells [6]. Beyond the retina, the expression pattern of CB1R has been observed in the dorsal lateral geniculate nucleus [8] and primary visual cortex [9] of primates.

Most of our knowledge on the role of cannabinoids in human vision comes from reports, anecdotes, and studies with cannabis consumers (for review see [10]). Besides the well-known “red eye” effect (vasodilation) of marijuana and reduction of intraocular pressure (IOP) [11–13], the functional effects of endocannabinoids on the visual system are not yet well defined [14]. Nevertheless, the administration of cannabinoids produces some known alterations in the human visual system. Indeed, case studies suggested the existence of cannabis-mediated visual effects in humans, particularly an increase in glare recovery at low contrast [15], a reduction in Vernier and Snellen acuities [16, 17], improvement in night vision [18, 19], blurred vision [20], changes in color discrimination, and an increase in photosensitivity [21]. Most of the latter (psychophysical) effects may have a retinal component, which might be due to neurochemical changes induced by the retinal endocannabinoid system. Indeed, many physiological effects of cannabinoids were reported for every retinal cell type in bovines, guinea pigs, rodents, and fishes (for review see [10, 22]). In the bovine retina, the activation of CB1R increases monoamine oxidase [23]. In the guinea pig retina, stimulation of CB1R results in the inhibition of dopamine release [24], and in the rat retina, the activation of cannabinoid receptors modulates [35S] GTP*γ* S-binding and voltage-dependent membrane currents in photoreceptors, bipolar cells, and ganglion cells [3, 25–28]. In addition, cannabinoid agonists increase the cone response to light offset in the goldfish retina [29].

The electroretinogram (ERG) is a useful tool for assessing retinal function by measuring the electrical responses of all populations of retinal cells, mainly photoreceptors (cones and rods), bipolar cells, amacrine cells, and Müller cells [30–32]. The ERG waves include two main components: the negative amplitude (a-wave) and the positive one (b-wave). Traditionally, the a-wave reflects the response of rods and cones to light [33, 34]. The generation of the b-wave, the second major component of the ERG, is attributed to the inner retina, mainly the depolarization of bipolar and Müller cells [30–32, 35–39]. Specific stimuli and recording environments are selected to isolate the components of the ERG and target particular populations of retinal cells. For instance, rod function is assessed in dark-adapted eyes, under scotopic conditions, while cone responses are better assessed with high intensity flashes, under photopic conditions [38]. In this study, we investigated the changes in normal retinal function as measured by electroretinography in adult vervet monkeys after blockade of CB1R or CB2R by their antagonists AM251 and AM630, respectively.

## 2. Material and Methods

### 2.1. Choice of Species

Vervet monkeys are becoming the preferred animal model used in biomedical research second only to the rhesus macaque [41]. Vervets are very similar in physiology and behavior to macaques, and they are more accessible and disease-free with less health and safety risks. Vervet monkeys have a foveal binocular vision with a high cone density that decreases with eccentricity, trichromatic color vision, and a six-layered dorsal lateral geniculate nucleus [42, 43]. Recently, we have standardized a noninvasive, painless ERG method for vervet monkeys [40] that showed highly comparable recordings to macaques [44] and humans [45].

### 2.2. Subjects

Sixteen vervet monkeys (*Chlorocebus sabaeus*) were tested in this study. Six of those monkeys were injected with AM251, and another six were injected with AM630. An additional 4 monkeys were injected with the vehicle (DMSO) used to dilute of our antagonists in order to provide control values. The animals were fed with primate chow (Harlan Teklad High Protein Monkey Diet; Harlan Teklad, Madison, WI, USA) and fresh local fruits, with water available* ad libitum.* All experiments were performed according to the guidelines of the Canadian Council on Animal Care (CCAC) and the Association for Research in Vision and Ophthalmology (ARVO) Statement for the Use of Animals in Ophthalmic and Vision Research. The experimental protocol was also reviewed and approved by the local Animal Care and Use Committee (University of Montreal, protocol # 14-007) and the Institutional Review Board of the Behavioral Science Foundation. None of the animals were sacrificed for this study.

### 2.3. Animal Preparation for ERG Recordings

All procedures were in accordance with the standard protocol of electroretinography in vervet monkeys [40]. Briefly, all animals received an intramuscular injection of ketamine (10 mg/kg; Troy Laboratories, Glendenning, New South Wales, Australia) and xylazine (1 mg/kg; Lloyd Laboratories, Shenandoah, IA, USA) to maintain an adequate level of sedation that prevents the animals from moving and blinking. This drug mixture has no effect on the ERG recordings [46]. With 1% tropicamide (Mydriacyl) and 2.5% phenylephrine hydrochloride (Mydfrin) (Alcon Laboratories, Fort Worth, TX, USA), the pupils were fully dilated (approximately 9 mm in diameter), with the accommodation paralyzed. The cornea was anesthetized with 0.5% proparacaine hydrochloride (Alcaine; Alcon Laboratories, Fort Worth, TX, USA). To prevent corneal drying, the eyes were moisturized frequently with 2.5% methylcellulose (Gonak; Akorn, Inc., Buffalo Grove, IL, USA). Body temperature was maintained between 36.5°C and 38°C with a heating pad. After a recording session that lasted about 2 hours, the animals were sent back to their prior natural settings after a recovery period in isolation.

### 2.4. Intravitreal Injection

The CB1R antagonist AM251 was purchased from Cayman Chemicals (Ann Arbor, MI, USA). The CB2R antagonist AM630 was purchased from Tocris (Tocris Bioscience, Ellisville, MO, USA). Both antagonists were diluted in DMSO under sterile conditions. Assuming no leakage, the final concentration was 1.5% v/v for DMSO, 0.01 mg/*μ*L for AM251, and 0.003 mg/*μ*L for AM630. To factor out any effects of the vehicle (DMSO), we subtracted the ERG recordings of the DMSO-injected animals from the ERG recordings of the drug injected animals. In this way, the effects that we report are only those above and beyond effects of the vehicle. After inspection and examination of the eyes, the cornea was cleaned with 5% povidone-iodine solution for 45 seconds. A drop of the topical anesthetic, proparacaine, was then applied over the injection site. The conjunctival and corneal surfaces were further moistened with methylcellulose (Moisture Eyes, Bausch & Lomb Canada, Vaughan, ON, Canada). The cornea was protected with sterile coatings while placing the Barraquer eye speculum (1.75 inches, 10 mm wide small blades). A total of 50 *μ*L of drug solution was injected 2 mm posterior to the corneal limbus into the vitreous cavity. Upon removal of the needle, the injection site was compressed for about one minute using a sterile cotton swab to avoid reflux. The back of the eye was inspected using an ophthalmoscope before and after the intravitreal injection to verify the integrity of the retina. No substantial differences were observed in intraocular pressure before and after the intravitreal administration. As a follow-up, the animals' eyes were checked every day for seven days following injection, and a topical antibiotic ointment was administered (Tobrex, 0.3% tobramycin ophthalmic ointment, Alcon Canada, Mississauga, Canada).

### 2.5. Visual Stimulation

Full-field stimulation was produced by a Ganzfeld light source (UTAS E-3000 electrophysiology equipment; LKC Technologies, Inc., Gaithersburg, MD, USA) that was placed in front of the animal's face. The ERGs were evoked by <5 ms white flashes delivered in full-field conditions. Xenon flash luminance of 2.5 to 800 cd·s·m^−2^ (0 dB to 20 dB in LKC units) was used for photopic recordings and LED flash luminance of 2.5 × 10^−5^ to 6 cd·s·m^−2^ (−40 dB to 4 dB in LKC units) for scotopic recordings. For light-adapted ERGs a steady background-adapting field (30 cd·m^−2^) was presented inside the Ganzfeld to saturate the rod system. Dark adaptation lasted approximately 20 minutes. Interstimulus intervals of at least 20 seconds were used at high intensities in the dark-adapted eyes. Flash intensities and background luminance were calibrated using a research radiometer (IL1700 Photometer, International Light Inc., Newburyport, MA, USA) with a SED033 detector placed at 36 cm from the source.

### 2.6. ERG Recording

**Figure 1:**
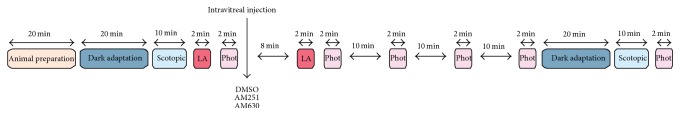
A schematic procedure illustrating a typical ERG recording session for testing ERG changes following an intravitreal injection in vervet monkeys (modified from [40]). LA, light adaptation; Phot, photopic.

All ERG procedures followed the ISCEV guidelines and the recently published standardized ERG protocol of vervet monkeys [40]. ERG responses were recorded separately between corneal contact lens electrodes (Jet electrodes, Diagnosys LLC, Lowell, MA, USA) lying across the center of the cornea of each eye. The jet electrodes were equipped with four small posts on the convex surface in order to keep the eyelids open. Reference and ground gold disc electrodes (model F-E5GH; Grass Technologies, Astro-Med, Inc., West Warwick, RI, USA) were, respectively, placed to the external canthi and forehead with adhesive paste (Ten20 conductive EEG paste, Kappa Medical, Prescott, AZ, USA). For the analysis of the waveforms, the a-wave amplitude was measured from the baseline to the trough of the a-wave. The amplitude of the b-wave was measured from the trough of the a-wave to the peak of the b-wave. The peak latency was defined from the onset of the flash to the trough or peak. Baselines and postinjection photopic amplitudes and latencies were calculated as averages to minimize the noise inherent in the ERG signals and improve power, allowing for robust parametric statistical analysis. Since there was only one baseline recording of the injected eye, the baseline value was calculated from an average across both eyes (when available) since ERGs do not vary considerably across eyes [47]. For the postinjection values, we had several recordings from the injected eye (one every 10 minutes for 40 minutes). Visual inspection revealed the peak effect to be present at both the 30 and 40 minutes of postinjection recordings. These were therefore averaged to obtain postinjection values for each intensity flash. Retinal response diagrams were drawn using Adobe Illustrator and processed in Adobe InDesign (Adobe Systems Canada, Ottawa, ON, Canada, software version CS5). The recording protocol for assessing the effect of the drugs is summarized in Figure 1.

### 2.7. Statistical Analysis

The absolute trough (a-wave) and peak (b-wave) of the ERG curves were obtained at each light intensity value. When the ERG curve for low light intensities (<−2 log cd·s·m^−2^) did not return to baseline 350 ms after the stimulus, the amplitudes of the a- and b-wave were corrected to account for the baseline shift. When no a-wave, or no wave at all, was detected, an amplitude of 0 was given and the latency was left blank for that specific stimulus intensity. Outliers (±2.5 SD) were removed (<2% overall). Postinjection amplitudes and latencies were expressed as percent of change from preinjection amplitudes (postinjection minus preinjection, divided by preinjection). The delta change percentages of injecting AM251 and AM630 were then subtracted from the delta change percent for the control injection, the vehicle DMSO. Thus, positive normalized effects indicate an increase as a result of injecting the drug, greater than the change that results from injecting the vehicle alone. To assess the statistical significance of the observed increase, we analyzed the amplitudes of the a- and b-waves using General Estimating Equations (GEE) with flash intensity as within subject factor, because each monkey was repeatedly measured (at each flash intensity). These main effects, and their interaction, were used to estimate the normalized effects of retinal injection of AM251 and AM630 on retinal function. Significant effects were followed up, when appropriate, with pairwise comparisons, significant values indicated with stars in the relevant figures.

## 3. Results

### 3.1. Retinal Function in Photopic Conditions

**Figure 2:**
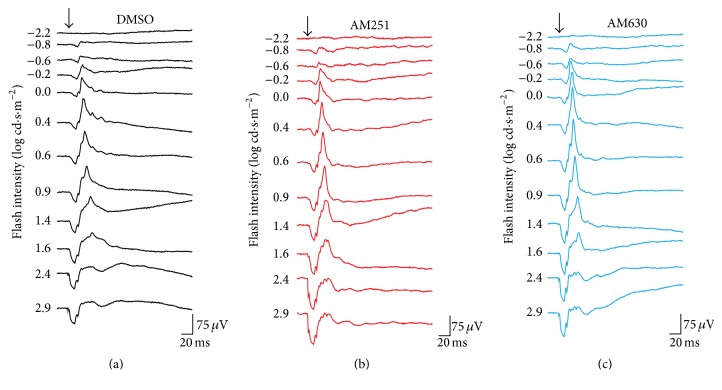
Raw photopic ERGs in the different drug injection groups. Representative ERGs recorded after intravitreal injection of DMSO (a), AM251 (b), or AM630 (c). ERG recordings of each treated animal were established by presenting progressively brighter flashes (top to bottom). Horizontal calibration, 20 ms; vertical calibration, 75 *μ*V.

Retinal function was evaluated using electroretinography in light-adapted conditions following injection of the vehicle DMSO, the CB1R antagonist AM251, or the CB2R antagonist AM630 (Figure 2).

### 3.2. Photopic b-Wave

**Figure 3:**
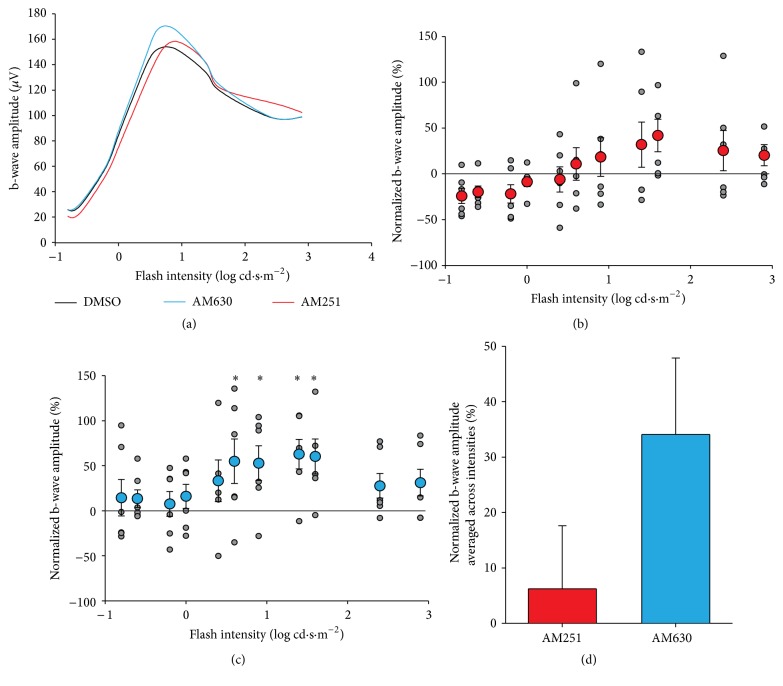
Photopic b-wave amplitudes. (a) Amplitudes of photopic ERG b-waves plotted as a function of flash intensities. (b) Scatter plot for normalized b-wave amplitude as a function of flash intensity in AM251-injected monkeys. Grey points indicate raw values; red data points with error bars indicate the mean and standard error of the mean. (c) Scatter plot and linear regression for normalized b-wave amplitude as a function of flash intensity in AM630-injected monkeys. Grey points indicate raw values; blue data points with error bars indicate the mean and standard error of the mean. (d) Main effect of average amplitudes across intensities in AM251 (red) or AM630 (blue) groups. ^*∗*^ *p* < .05.

Our results show that the amplitudes of the b-wave after injection maintained a normal photopic hill shape indicating that the functional integrity of the retina was not impaired (Figure 3(a)). GEE analysis revealed a significant main effect of flash intensity (*p* < .001), and a significant interaction between the flash intensity and injection group (*p* = .001). The interaction was followed up with pairwise comparisons. AM251 was not significantly different from DMSO at any of the flash intensities (Figure 3(b)). In contrast, AM630 caused a significant increase in amplitude, relative to the control injection, across several flash intensities, from 0.6 to 1.6 log cd·s·m^−2^ (0.6: 55% increase in amplitude relative to the control, *p* = .041; 0.9: 53% increase, *p* = .038; 1.4: 63% increase, *p* = .003; 1.6: 60% increase, *p* = .011; significant effects indicated with *∗* in Figure 3(c)). The main effect of AM251 is, on average, a 6% increase, which is not significantly different from the vehicle alone, represented by zero on the *y*-axis of Figures 3(b)–3(d) (*p* = .713). The main effect of AM630, averaged across all flash intensities, is a nonsignificant, but trending, 34% increase in responsiveness of the retina compared to the vehicle alone (*p* = .067, Figure 3(d)). Latencies were also analyzed with the same GEE model and the interaction (*p* < .05) was followed up as above. Pairwise comparisons revealed no significant differences, between the drugs and the vehicle, at any of the flash intensities (not shown).

### 3.3. Photopic a-Wave

**Figure 4:**
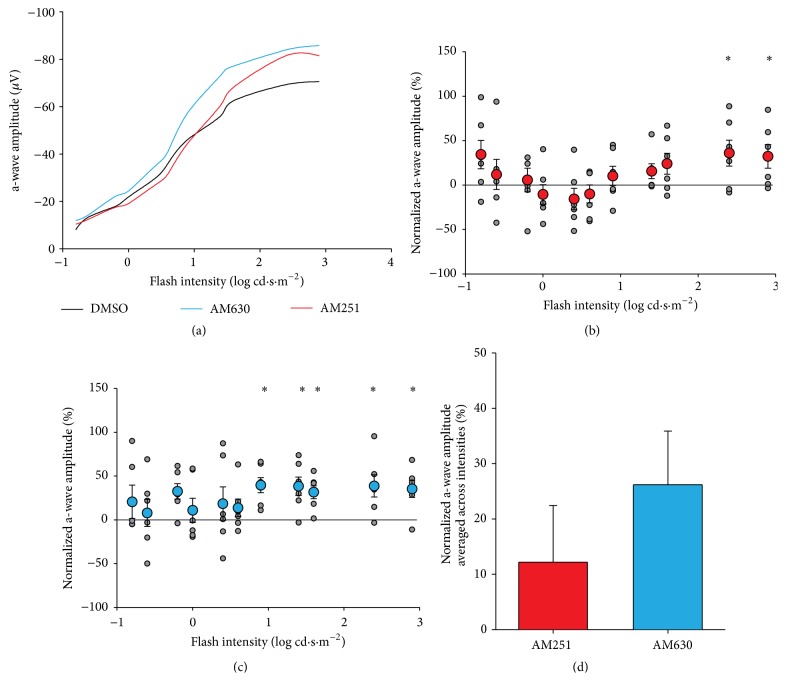
Photopic a-wave amplitudes. (a) Amplitudes of photopic ERG a-waves plotted as a function of flash intensities. (b) Scatter plot for normalized a-wave amplitude as a function of flash intensity in AM251-injected monkeys. Grey points indicate raw values; red data points with error bars indicate the mean and standard error of the mean. (c) Scatter plot and linear regression for normalized a-wave amplitude as a function of flash intensity in AM630-injected monkeys. Grey points indicate raw values; blue data points with error bars indicate the mean and standard error of the mean. (d) Main effect of average amplitudes across intensities in AM251 (red) or AM630 (blue) groups. ^*∗*^ *p* < .05.

Our results show that, after injection, the amplitude of the a-wave followed the normal curve (Figure 4(a)). The effect of the drugs was, however, quite different from the vehicle. GEE analysis revealed a significant main effect of flash intensity (*p* < .001), and a significant interaction between the flash intensity and drug group (*p* < .001). This interaction indicates that the effect of the drugs was not the same across all flash intensities. The interaction was followed up with pairwise comparisons. AM251 caused a significantly higher amplitude than DMSO at the highest flash intensities of 2.4 and 2.9 log cd·s·m^−2^ (2.4: 36% increase in amplitude relative to the vehicle, *p* = .040; 2.9: 32% increase, *p* = .038; significant effects indicated with *∗* in Figure 4(b)). For its part, AM630 caused a significant increase in amplitude, relative to the control injection, across a larger set of flash intensities, from 0.9 to 2.9 log cd·s·m^−2^ ((0.9: 30% increase, *p* < .001; 1.4: 38% increase, *p* = .002; 1.6: 32% increase, *p* = .001; 2.4: 39% increase, *p* = .015; 2.9: 35% increase, *p* = .006), significant effects indicated with *∗* in Figure 4(c)). AM251 caused an increase in the a-wave amplitude only at the highest flash intensities, while AM630 increases the a-wave amplitude across a wider set of flash intensities. The main effect of AM251 is, on average, a 12% increase, which is not significant relative to the vehicle alone (*p* = .428). Conversely, the main effect of AM630, averaged across all flash intensities, is a nonsignificant but trending 26% increase in the responsiveness of the retina compared to the vehicle alone (*p* = .080, Figure 4(d)). Latencies were also analyzed with the same GEE model and the interaction (*p* < .05) was followed up as above. Pairwise comparisons at each intensity value revealed no significant differences between the drugs and the vehicle (not shown).

### 3.4. Retinal Function in Scotopic Condition

**Figure 5:**
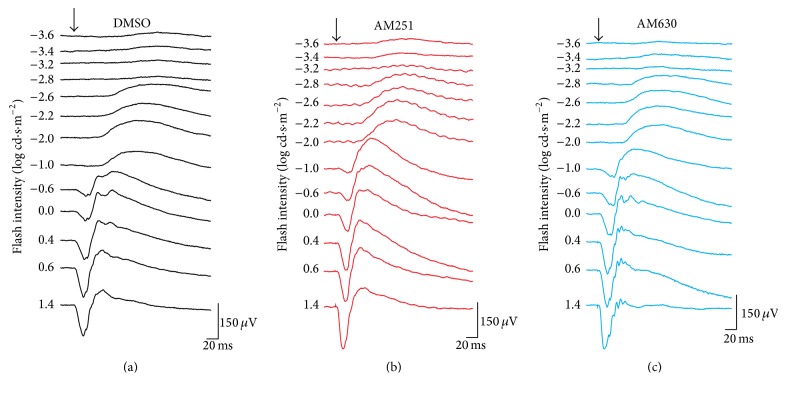
Raw scotopic ERGs in the different drug injection groups. Representative ERGs recorded after intravitreal injection of DMSO (a), AM251 (b), or AM630 (c). ERG recordings of each treated animal were established by presenting progressively brighter flashes (top to bottom). Horizontal calibration, 20 ms; vertical calibration, 75 *μ*V.

To assess the effect of the cannabinoid receptor antagonists in scotopic conditions, ERG responses of the dark-adapted retina were also registered after administration of DMSO, AM251, or AM630. The ERG tracings maintained their normal shape following injection. However, the amplitudes of the b-wave were increased for both treatment groups, while the drugs did not reliably alter the pattern of the a-waves (Figure 5).

### 3.5. Scotopic b-Wave

**Figure 6:**
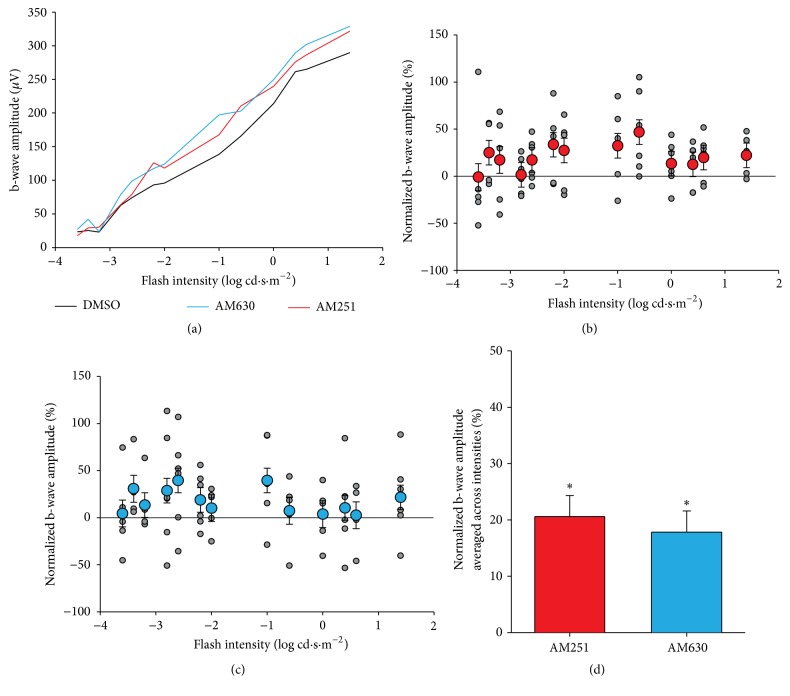
Scotopic b-wave amplitudes. (a) Amplitudes of scotopic ERG b-waves plotted as a function of flash intensities. (b) Scatter plot for normalized b-wave amplitude as a function of flash intensity in AM251-injected monkeys. Grey points indicate raw values; red data points with error bars indicate the mean and standard error of the mean. (c) Scatter plot and linear regression for normalized b-wave amplitude as a function of flash intensity in AM630-injected monkeys. Grey points indicate raw values; blue data points with error bars indicate the mean and standard error of the mean. (d) Main effect of average amplitudes across intensities in AM251 (red) or AM630 (blue) groups. ^*∗*^ *p* < .05.

After injection, the amplitude of the scotopic b-waves had the normal shape: increasing amplitudes for increasing flash intensities (Figure 6(a)). The effect of the drugs was, however, quite different from the vehicle. GEE analysis revealed a significant main effect of injection group (*p* < .001). There was no main effect of flash intensity (*p* = .842) nor was there an interaction of group with flash intensity (*p* = .953). Due to a lack of interaction, pairwise comparisons at each flash intensity were not justified, but mean and standard errors are plotted in Figure 6(b) (AM251) and Figure 6(c) (AM630). Following up on the main effect of drug, pairwise comparisons between groups revealed significantly higher amplitudes following the injection of AM251 compared to the vehicle alone (20% increase, *p* < .001) and a similar increase in amplitude following injection of AM630 (18% increase, *p* = .002) (Figure 6(d)). The difference between these two drugs was not significant (*p* = .596). Latencies had the same pattern of effect as the amplitudes. Both pharmacological agents led to a significant increase in the latency relative to the vehicle (AM251: 8%, *p* = .036; AM630: 12%, *p* = .001). No interactions were present (not shown).

### 3.6. Scotopic a-Wave

**Figure 7:**
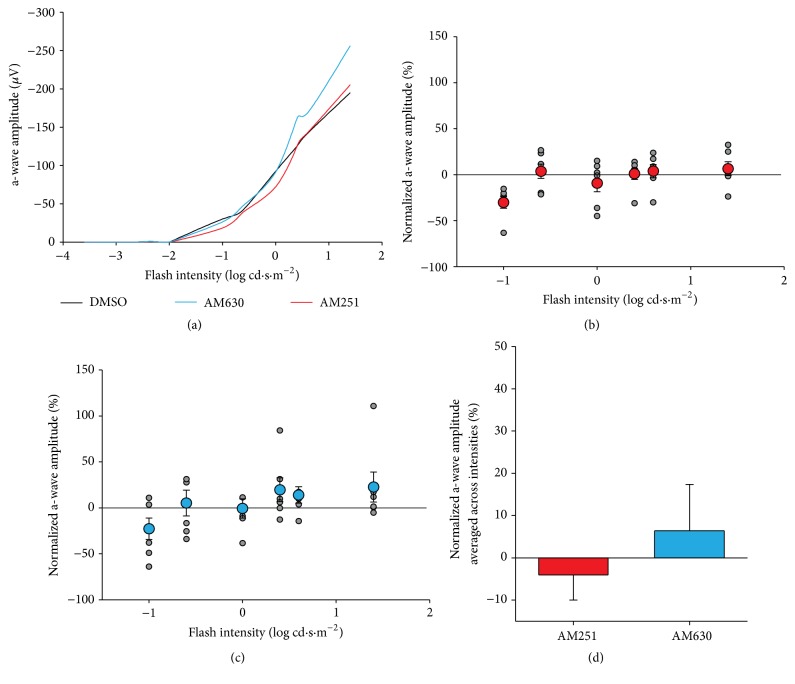
Scotopic a-wave amplitudes. (a) Amplitudes of scotopic ERG a-waves plotted as a function of flash intensities. (b) Scatter plot for normalized a-wave amplitude as a function of flash intensity in AM251-injected monkeys. Grey points indicate raw values; red data points with error bars indicate the mean and standard error of the mean. (c) Scatter plot for normalized a-wave amplitude as a function of flash intensity in AM630-injected monkeys. Grey points indicate raw values; blue data points with error bars indicate the mean and standard error of the mean. (d) Main effect of average amplitudes across intensities in AM251 (red) or AM630 (blue) groups. Error bars represent standard error or the mean.

After injection, the amplitude of the scotopic a-wave had the normal shape: increasing amplitudes for increasing flash intensities, beginning at −1 log cd·s·m^−2^ (Figure 7(a)). Therefore, the statistical analysis for the scotopic a-wave only involved the values obtained from the flashes at −1 to 1.4 log cd·s·m^−2^. GEE analysis revealed a significant main effect of flash intensity (*p* = .001) and a significant interaction between the intensity of the flash and the drug injected (*p* < .001). The interaction was followed up with pairwise comparisons, between the drugs and the vehicle alone, at each flash intensity value. This revealed no significant differences, between the drugs and the vehicle, at any of the intensities (Figures 7(b) and 7(c)). Thus, while the two agonists cause varied effects at the different flash intensities, the effects of each drug relative to the vehicle alone, at a given flash intensity, were not reliable enough to be significant. The overall effect of AM251 and AM630, averaged across the flash intensities, was not significantly different from the vehicle alone (Figure 7(d)). Latencies were also analyzed with the same GEE model and the interaction (*p* < .05) was followed up as above. Pairwise comparisons revealed one significant difference at flash intensity 0.6; following injection of AM251, the latency to peak was 21% slower compared to the injection of the vehicle alone (*p* = .011, not shown).

## 4. Discussion

The purpose of this study was to determine the role of cannabinoid receptors CB1 and CB2 in the normal monkey retina. The abundance of CB1R and CB2R expression in the retina already pointed to an important role of these receptors in normal vision. We analyzed changes in photopic and scotopic ERG responses after blocking these receptors with their respective antagonists. The experimental design used DMSO as the control, rather than preinjection values, to control for any effects of the vehicle. We demonstrated that, in photopic conditions, only the blockade of CB2R increased the amplitude of the b-wave, above the standard flash intensity value, while blocking CB1R or CB2R increased the amplitude of the a-wave, at high flash intensity values. In scotopic conditions, however, blockade of either CB1R or CB2R increased only the amplitude of the b-wave irrespective of flash intensity.

### 4.1. Photopic Condition

**Figure 8:**
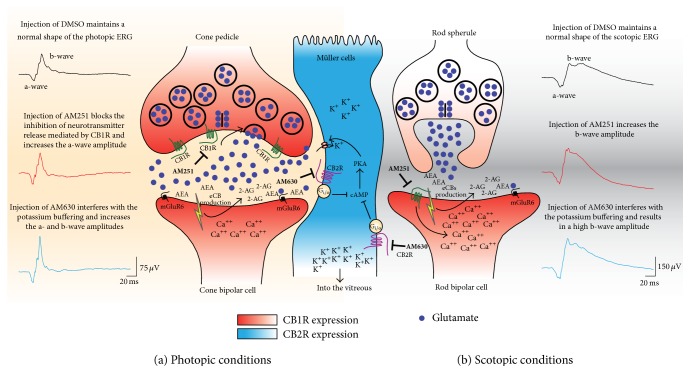
Schematic illustration of the proposed mechanisms underlying the actions of AM251 and AM630 in the monkey retina, as revealed by electroretinography under photopic (a) and scotopic (b) conditions. (See Discussion for details.)

The amplitude of the main component of the ERG, the photopic b-wave, represents primarily the activation of depolarization ON-bipolar cells measured as a positive retinal potential on the corneal surface [36, 48–50]. In addition, the b-wave is attributed to the interaction of ON-bipolar cells and Müller glial cells [36, 37]. In the vervet monkey, CB1R is expressed mainly in cones and in the other retinal components, while CB2R is exclusively present in the glial Müller cells, leading to a complementary relationship between neurons and glia regarding endocannabinoid action [6]. The light-induced potassium increase in the outer and inner plexiform layers' cells, which are depolarized by light stimulation, modifies the Müller cell membrane potential thereby generating electrical responses [51]. Müller cells, via KCNJ10 (K_IR_4.1) channels and potassium siphoning of the excess potassium ions into the vitreous [52, 53], control the light-mediated potassium increase in retinal extracellular space [54]. The depolarization of the Müller cells contributes to the ERG b-wave through the buffering of potassium channels [35, 55, 56]. Thus, the blockade of potassium channels should result in a decrease of the ERG b-wave [37]. Our results revealed a significant increase of the photopic b-wave amplitude following the blockade of CB2R, which supports our previously proposed model [6]. CB2R coupled to G_i/o_ decreases cAMP levels and the PKA activity [57]. PKA is a positive modulator of potassium channels and therefore, the blockade of CB2R via an increase of PKA activity will increase the activity of K^+^ channels in Müller cells, and thus an increased photopic b-wave amplitude (Figure 8). It may also be possible that AM630 affected the OFF cone pathway that originates from the dendritic contacts of bipolar cells with cones, which could partially explain the increase of the photopic b-wave amplitude only at the middle intensity flash values [58]. Another potential interpretation is that since CB2R is not expressed on cones [6], AM630 may have modulated other non-CB2 receptors located on cone photoreceptors.

The a-wave measured under photopic conditions represents cone function. Stimulation of cones by light inhibits retinal dark currents through phototransduction signals that take place in the cone outer segments as seen in the a-wave of the ERG. The early portion of the a-wave represents the activity of the cone photoreceptors [59, 60], while the later portion reflects the contribution of hyperpolarizing bipolar cells, proximal amacrine cells, and ganglion cells [61–63]. Stimulation of cones may activate the CB1R in its pedicles [5], which in turn leads to the inhibition of glutamate release in the synaptic cleft. Blocking CB1R will therefore result in the increase in glutamate release. This increase mimics the effect of a bright light that contributes to larger amplitude of the photopic a-wave. Blocking CB2R on the other hand has an even larger effect on the amplitude of the a-wave, which can be explained by a similar mechanism that involves additional potassium buffering by Müller cells [6] (Figure 8(a)). Other receptors that contribute to the photopic b-wave may also explain how AM630 could affect the photopic a-wave. Indeed, the increase of the photopic b-wave might cause a large change in the a-wave (a result of cone modulation), which would be independent of the action of Müller cells.

### 4.2. Scotopic Condition

The a-wave measured under scotopic conditions represents rod function. In the dark-adapted retina, blockade of either CB1R or CB2R had no significant effect on the scotopic a-wave. This null effect can be explained by the small quantity of CB1R expressed in the rod spherules in primates [3, 5]. In contrast, the large quantity of putative cannabinoid receptor (GPR55), found exclusively in rods [64], has a significant effect on the scotopic ERG [65]. It has been reported that AM251 could be also a GPR55 agonist [66]. Thus, we cannot rule out that the increase of the scotopic b-wave amplitude following the injection of AM251 might be due to GPR55 activity. However, CB1R is found in large quantities in rod bipolar cells [5] and, in conjunction with CB2R in Müller cells [6], likely contributes to the large increase of the scotopic b-wave amplitude. Differential effects between CB1R and CB2R might be explained by the nature of the ion channels involved. The potassium buffering role of Müller cells leads to the increase of the scotopic b-wave following CB2R blockade. The calcium increase in postsynaptic rod bipolar cells results from CB1R blockade (see Figure 8(b)). Since CB1R agonists induce a reduction in the amplitude of calcium channel currents in retinal bipolar cells [3], it is not surprising, as shown here, that the CB1R antagonist AM251 had the opposite effect: mainly, an increase in rod bipolar cells activity.

## 5. Conclusion

These findings might be helpful for the development of new pharmacological targets for the treatment of retinal intoxication [67, 68] and diseases [69]. These retinal pathologies are generally associated with a decrease in the amplitude of the electroretinographic waves. We show here that pharmacological agents that block the retinal cannabinoid receptors can induce an increase in the amplitude of the ERG response profiles. Manipulating the endocannabinoid system might therefore serve as a therapy to restore normal vision and protect the retina.

## References

[1] Cécyre B., Zabouri N., Huppé-Gourgues F., Bouchard J.-F., Casanova C. (2013). Roles of cannabinoid receptors type 1 and 2 on the retinal function of adult mice. *Investigative Ophthalmology and Visual Science*.

[2] López E. M., Tagliaferro P., Onaivi E. S., López-Costa J. J. (2011). Distribution of CB2 cannabinoid receptor in adult rat retina. *Synapse*.

[3] Straiker A., Stella N., Piomelli D., Mackie K., Karten H. J., Maguire G. (1999). Cannabinoid CB1 receptors and ligands in vertebrate retina: localization and function of an endogenous signaling system. *Proceedings of the National Academy of Sciences of the United States of America*.

[4] Yazulla S., Studholme K. M., McIntosh H. H., Deutsch D. G. (1999). Immunocytochemical localization of cannabinoid CB1 receptor and fatty acid amide hydrolase in rat retina. *Journal of Comparative Neurology*.

[5] Bouskila J., Burke M. W., Zabouri N., Casanova C., Ptito M., Bouchard J.-F. (2012). Expression and localization of the cannabinoid receptor type 1 and the enzyme fatty acid amide hydrolase in the retina of vervet monkeys. *Neuroscience*.

[6] Bouskila J., Javadi P., Casanova C., Ptito M., Bouchard J.-F. (2013). Müller cells express the cannabinoid CB2 receptor in the vervet monkey retina. *Journal of Comparative Neurology*.

[7] Zabouri N., Bouchard J.-F., Casanova C. (2011). Cannabinoid receptor type 1 expression during postnatal development of the rat retina. *Journal of Comparative Neurology*.

[8] Javadi P., Bouskila J., Bouchard J.-F., Ptito M. (2015). The endocannabinoid system within the dorsal lateral geniculate nucleus of the vervet monkey. *Neuroscience*.

[9] Eggan S. M., Lewis D. A. (2007). Immunocytochemical distribution of the cannabinoid CB1 receptor in the primate neocortex: a regional and laminar analysis. *Cerebral Cortex*.

[10] Schwitzer T., Schwan R., Angioi-Duprez K. (2015). The cannabinoid system and visual processing: a review on experimental findings and clinical presumptions. *European Neuropsychopharmacology*.

[11] Flom M. C., Adams A. J., Jones R. T. (1975). Marijuana smoking and reduced pressure in human eyes: drug action or epiphenomenon?. *Investigative Ophthalmology*.

[12] Green K. (1979). The ocular effects of cannabinoids. *Current Topics in Eye Research*.

[13] Porcella A., Casellas P., Gessa G. L., Pani L. (1998). Cannabinoid receptor CB1 mRNA is highly expressed in the rat ciliary body: implications for the antiglaucoma properties of marihuana. *Molecular Brain Research*.

[14] Laprevote V., Schwitzer T., Giersch A., Schwan R. (2015). Flash electroretinogram and addictive disorders. *Progress in Neuro-Psychopharmacology and Biological Psychiatry*.

[15] Adams A. J., Brown B., Haegerstrom-Portnoy G., Flom M. C., Jones R. T. (1978). Marijuana, alcohol, and combined drug effects on the time course of glare recovery. *Psychopharmacology*.

[16] Adams A. J., Brown B., Flom M. C., Jones R. T., Jampolsky A. (1975). Alcohol and marijuana effects on static visual acuity. *American Journal of Optometry and Physiological Optics*.

[17] Kiplinger G. F., Manna J. E., Rodda B. E., Forney R. B. (1971). Dose-response analysis of the effects of tetrahydrocannabinol in man. *Clinical Pharmacology and Therapeutics*.

[18] Merzouki A., Mesa J. M. (2002). Concerning kif, a *Cannabis sativa* L. preparation smoked in the Rif mountains of northern Morocco. *Journal of Ethnopharmacology*.

[19] Russo E. B., Merzouki A., Mesa J. M., Frey K. A., Bach P. J. (2004). Cannabis improves night vision: a case study of dark adaptometry and scotopic sensitivity in kif smokers of the Rif mountains of northern Morocco. *Journal of Ethnopharmacology*.

[20] Noyes R., Brunk S. F., Avery D. H., Canter A. (1975). The analgesic properties of delta-9-tetrahydrocannabinol and codeine. *Clinical Pharmacology and Therapeutics*.

[21] Dawson W. W., Jimenez Antillon C. F., Perez J. M., Zeskind J. A. (1977). Marijuana and vision—after ten years' use in Costa Rica. *Investigative Ophthalmology and Visual Science*.

[22] Yazulla S. (2008). Endocannabinoids in the retina: from marijuana to neuroprotection. *Progress in Retinal and Eye Research*.

[23] Gawienowski A. M., Chatterjee D., Anderson P. J., Epstein D. L., Grant W. M. (1982). Effect of Δ9-tetrahydrocannabinol on monoamine oxidase activity in bovine eye tissues, in vitro. *Investigative Ophthalmology and Visual Science*.

[24] Schlicker E., Timm J., Gothert M. (1996). Cannabinoid receptor-mediated inhibition of dopamine release in the retina. *Naunyn-Schmiedeberg's Archives of Pharmacology*.

[25] Fan S.-F., Yazulla S. (1999). Suppression of voltage-dependent K^+^ currents in retinal bipolar cells by ascorbate. *Visual Neuroscience*.

[26] Fan S.-F., Yazulla S. (2003). Biphasic modulation of voltage-dependent currents of retinal cones by cannabinoid CB1 receptor agonist WIN 55212-2. *Visual Neuroscience*.

[27] Straiker A., Sullivan J. M. (2003). Cannabinoid receptor activation differentially modulates ion channels in photoreceptors of the tiger salamander. *Journal of Neurophysiology*.

[28] Yazulla S., Studholme K. M., McIntosh H. H., Fan S.-F. (2000). Cannabinoid receptors on goldfish retinal bipolar cells: electron-microscope immunocytochemistry and whole-cell recordings. *Visual Neuroscience*.

[29] Struik M. L., Yazulla S., Kamermans M. (2006). Cannabinoid agonist WIN 55212-2 speeds up the cone response to light offset in goldfish retina. *Visual Neuroscience*.

[30] Frishman L. J., Ryan S. J., Schachat A. P., Wilkinson C. P., Hinton D. R., Sadda S. R., Wiedemann P. (2013). Electrogenesis of the electroretinogram. *Retina*.

[31] Frishman L. J., Heckenlively J. R., Arden G. B. (2006). Origins of the electroretinogram. *Principles and Practice of Clinical Electrophysiology of Vision*.

[32] Frishman L. J., Wang M. H., Levin L. A., Nilsson S. F. E., Hoeve J. V., Wu S., Kaufman P. L., Alm A. (2011). Electroretinogram of human, monkey and mouse. *Adler's Physiology of the Eye*.

[33] Armington J. C., Johnson E. P., Riggs L. A. (1952). The scotopic A-wave in the electrical response of the human retina. *The Journal of Physiology*.

[34] Robson J. G., Frishman L. J. (2014). The rod-driven a-wave of the dark-adapted mammalian electroretinogram. *Progress in Retinal and Eye Research*.

[35] Miller R. F., Dowling J. E. (1970). Intracellular responses of the Müller (glial) cells of mudpuppy retina: their relation to b-wave of the electroretinogram. *Journal of Neurophysiology*.

[36] Stockton R. A., Slaughter M. M. (1989). B-wave of the electroretinogram. A reflection of ON bipolar cell activity. *Journal of General Physiology*.

[37] Wen R., Oakley B. (1990). K^+^-evoked Müller cell depolarization generates b-wave of electroretinogram in toad retina. *Proceedings of the National Academy of Sciences of the United States of America*.

[38] Robson J. G., Frishman L. J. (1995). Response linearity and kinetics of the cat retina: the bipolar cell component of the dark-adapted electroretinogram. *Visual Neuroscience*.

[39] Hamilton R., Bees M. A., Chaplin C. A., McCulloch D. L. (2007). The luminance-response function of the human photopic electroretinogram: a mathematical model. *Vision Research*.

[40] Bouskila J., Javadi P., Palmour R. M., Bouchard J.-F., Ptito M. (2014). Standardized full-field electroretinography in the green monkey (*Chlorocebus sabaeus*). *PLoS ONE*.

[41] Jasinska A. J., Schmitt C. A., Service S. K. (2013). Systems biology of the vervet monkey. *ILAR Journal*.

[42] Boire D., Théoret H., Ptito M. (2001). Visual pathways following cerebral hemispherectomy. *Progress in Brain Research*.

[43] Herbin M., Boire D., Ptito M. (1997). Size and distribution of retinal ganglion cells in the St. Kitts green monkey (*Cercopithecus aethiops sabeus*). *Journal of Comparative Neurology*.

[44] Bee W. H. (2001). Standardized electroretinography in primates: a non-invasive preclinical tool for predicting ocular side effects in humans. *Current Opinion in Drug Discovery and Development*.

[45] McCulloch D. L., Marmor M. F., Brigell M. G. (2015). ISCEV standard for full-field clinical electroretinography (2015 update). *Documenta Ophthalmologica*.

[46] Nair G., Kim M., Nagaoka T. (2011). Effects of common anesthetics on eye movement and electroretinogram. *Documenta Ophthalmologica*.

[47] Rotenstreich Y., Fishman G. A., Anderson R. J., Birch D. G. (2003). Interocular amplitude differences of the full field electroretinogram in normal subjects. *British Journal of Ophthalmology*.

[48] Knapp A. G., Schiller P. H. (1984). The contribution of on-bipolar cells to the electroretinogram of rabbits and monkeys—a study using 2-amino-4-phosphonobutyrate (APB). *Vision Research*.

[49] Sieving P. A., Murayama K., Naarendorp F. (1994). Push-pull model of the primate photopic electroretinogram: a role for hyperpolarizing neurons in shaping the b-wave. *Visual Neuroscience*.

[50] Tian N., Slaughter M. M. (1995). Correlation of dynamic responses in the ON bipolar neuron and the*b*-wave of the electroretinogram. *Vision Research*.

[51] Dick E., Miller R. F., Bloomfield S. (1985). Extracellular K+ activity changes related to electroretinogram components. II. Rabbit (E-type) retinas. *Journal of General Physiology*.

[52] Newman E. A., Frambach D. A., Odette L. L. (1984). Control of extracellular potassium levels by retinal glial cell K+ siphoning. *Science*.

[53] Kofuji P., Ceelen P., Zahs K. R., Surbeck L. W., Lester H. A., Newman E. A. (2000). Genetic inactivation of an inwardly rectifying potassium channel (kir4.1 subunit) in mice: phenotypic impact in retina. *The Journal of Neuroscience*.

[54] Newman E., Reichenbach A. (1996). The Müller cell: a functional element of the retina. *Trends in Neurosciences*.

[55] Newman E. A. (1987). Distribution of potassium conductance in mammalian Müller (glial) cells: a comparative study. *The Journal of Neuroscience*.

[56] Steinberg R. H., Frishman L. J., Sieving P. A. (1991). Chapter 6 Negative components of the electroretinogram from proximal retina and photoreceptor. *Progress in Retinal Research*.

[57] Howlett A. C., Barth F., Bonner T. I. (2002). International Union of Pharmacology. XXVII. Classification of cannabinoid receptors. *Pharmacological Reviews*.

[58] Ueno S., Kondo M., Niwa Y., Terasaki H., Miyake Y. (2004). Luminance dependence of neural components that underlies the primate photopic electroretinogram. *Investigative Ophthalmology and Visual Science*.

[59] Hood D. C., Birch D. G. (1993). Human cone receptor activity: the leading edge of the a-wave and models of receptor activity. *Visual Neuroscience*.

[60] Hood D. C., Birch D. G. (1996). Assessing abnormal rod photoreceptor activity with the a-wave of the electroretinogram: applications and methods. *Documenta Ophthalmologica*.

[61] Bush R. A., Sieving P. A. (1994). A proximal retinal component in the primate photopic ERG a-wave. *Investigative Ophthalmology & Visual Science*.

[62] Friedburg C., Allen C. P., Mason P. J., Lamb T. D. (2004). Contribution of cone photoreceptors and post-receptoral mechanisms to the human photopic electroretinogram. *Journal of Physiology*.

[63] Robson J. G., Saszik S. M., Ahmed J., Frishman L. J. (2003). Rod and cone contributions to the a-wave of the electroretinogram of the macaque. *The Journal of Physiology*.

[64] Bouskila J., Javadi P., Casanova C., Ptito M., Bouchard J.-F. (2013). Rod photoreceptors express GPR55 in the adult vervet monkey retina. *PLoS ONE*.

[65] Bouskila J., Harrar V., Javadi P. Scotopic vision in the monkey is modulated by the G protein-coupled receptor 55. *Visual Neuroscience*.

[66] Pertwee R. G. (2007). GPR55: a new member of the cannabinoid receptor clan?. *British Journal of Pharmacology*.

[67] Hennekes R. (1982). Clinical ERG findings in ethambutol intoxication. *Graefe's Archive for Clinical and Experimental Ophthalmology*.

[68] Duncker G., Bredehorn T. (1996). Chloroquine-induced lipidosis in the rat retina: functional and morphological changes after withdrawal of the drug. *Graefe's Archive for Clinical and Experimental Ophthalmology*.

[69] Nilsson S. E. (1971). Human retinal vascular obstructions. A quantitative correlation of angiographic and electroretinographic findings. *Acta Ophthalmologica*.

